# Evaluation of the rapid, multi-country, parallel process, multi-tasking approach to startup of short-term technical assistance to improve service delivery in newborn and child health in the context of USAID’s Zika response in four Eastern and Southern Caribbean countries

**DOI:** 10.12688/f1000research.22814.2

**Published:** 2022-01-27

**Authors:** Bulbul Aumakhan, Astou Coly, Salwan Hager, Tamar Chitashvili, M. Rashad Massoud

**Affiliations:** 1 Independent Consultant, Laurel, MD, USA; 2 University Research Co., LLC, Chevy Chase, MD, 20815, USA; 3 USAID Applying Science to Strengthen and Improve Systems Project (ASSIST), University Research Co., LLC, Chevy Chase, MD, 20815, USA

**Keywords:** Rapid startup, Zika, emergency response, quality improvement, Eastern and Southern Caribbean, Antigua and Barbuda, Dominica, St. Kitts and Nevis, St. Vincent and the Grenadines

## Abstract

**Background:** In 2018, the USAID Applying Science to Strengthen and Improve Systems (ASSIST) Project started a new partnership with four Eastern and Southern Caribbean countries impacted by the Zika virus: Antigua and Barbuda, Dominica, St. Kitts and Nevis, and St. Vincent and the Grenadines.  The goal of the project was to provide short-term technical assistance (STTA) to strengthen the health systems’ capacity to detect newborns and young children potentially affected by Zika and to address their health needs.  To meet these objectives, ASSIST developed an innovative approach based on its existing model for service delivery improvement. This novel approach is known as Rapid, Multi-country, Parallel Process, Multi-tasking Approach for a Project Startup (RMPP-MAPS).  An evaluation was conducted to document the STTA startup activities, to identify enabling and constraining factors, and to capture lessons learned.

**Methods:** An external consultant conducted remote in-depth interviews with individuals involved in the startup using semi-structured interview guides and retrieved data from the review of project documents.

**Results: **Using RMPP-MAPS, the ASSIST Project successfully implemented the startup for complex STTA in four countries within less than four months, spanning mid-May to early September 2018. Project milestones included achieving buy-in from stakeholders, co-developing the technical scope and materials, and rapidly executing critical operational functions.  Dedicated project teams, country leaderships, and local champions were essential to overcoming the main challenges, which included a condensed timeframe, lack of in-country offices, and country-level factors such as a shortage of health care workers and a weak health infrastructure.

**Conclusions**: The RMPP-MAPS is a feasible and resource-efficient mechanism of interest to implementers, donors, and low and middle-income countries facing temporal and financial limitations to rapidly addressing public health priorities.

## Introduction

### Background

The rapid spread of the Zika virus (ZIKV) infection to countries in the Latin American and Caribbean (LAC) region in 2015 and 2016 and its association with serious health consequences such as microcephaly prompted the World Health Organization (WHO) to declare the ZIKV infection a public health emergency of international concern (PHEIC) on February 1, 2016 ^1^. Although the WHO declared an end to the PHEIC in November of the same year, the ongoing Zika transmission in the region as well as the potential for future outbreaks required continuous vigilance to improve health systems preparedness and capacity in prevention, surveillance, and management of emerging infectious diseases ^2– 4^.

In 2016, the United States Agency for International Development (USAID), the international community, and country governments began responding to the ZIKV epidemic in the LAC region ^5^. In 2017, the USAID Applying Science to Strengthen and Improve Systems (ASSIST) Project implemented by University Research Co., LLC (URC) in Chevy Chase, MD, started activities in the Dominican Republic, Ecuador, El Salvador, Guatemala, Honduras, Jamaica, Nicaragua, and Paraguay to strengthen Zika-related health services to deliver evidence-based, person-centered quality care with a focus on pregnant women, newborns, and women of reproductive age ^6^. In May 2018, USAID requested that ASSIST expand its efforts to four more countries in the Eastern and Southern Caribbean (ESC): Antigua and Barbuda, Dominica, St. Kitts and Nevis, and St. Vincent and the Grenadines. The request came in the last year of a three-year USAID Zika response plan in the LAC region. During this time, there was also a substantial decline in the number of Zika cases observed in the region. Therefore, the decision was made to design the effort as a one-year short-term technical assistance (STTA) activity aimed at improving detection and addressing the unique health needs of children potentially affected by Zika in the four countries. The STTA planned to achieve these objectives by strengthening newborn and well-baby care systems and early childhood development (ECD) programs in all functional health facilities providing childbirth services and well-baby care. These efforts were in line with national and USAID goals to improve public health emergency response and to strengthen the resilience of health systems to address future emergencies.

### Rapid Multi-Country, Parallel Process, Multi-Tasking Approach for Project Startup

Given the short timeline and overarching objectives, ASSIST developed and applied the Rapid, Multi-country, Parallel Process, Multi-tasking Approach to Project Startup (RMPP-MAPS) in the four countries. The RMPP-MAPS required simultaneous initiation and execution of multiple planning processes; including but not limited to, achieving buy-in from Ministries of Health and participating facilities, co-developing the workplan and sustainability strategy, developing technical content and operational approach, identifying essential needs of facility teams to implement the activity, and initiating the procurement processes to address the logistical needs of the activity.

### Basis and rationale for the RMPP-MAPS

The fundamental concept underlying ASSIST’s health systems improvement approach is understanding that “every system is perfectly designed to achieve exactly the results it achieves ^7^.” ASSIST views health systems as complex yet adaptive systems designed to undergo continuous reiterative processes of change and innovation. That process forms the basis of a six-component “Country Integrated Design” model (1-Improvement Design; 2-Implementation; 3-Sustainability; 4-Scale-up; 5-Institutionalization, and 6- Learning) that has been proven to effect positive outcomes over the three decades of USAID work (through the ASSIST and preceding projects) in the field of health system strengthening and quality improvement in health care services delivery ^8^. To achieve desired outcomes, all six components of the model must be integrated in planning improvement interventions. Thus, the process necessitates the engagement of all stakeholders throughout implementation. Depending on the scale of the problem and/or context the process can take up to several years. A typical model of ASSIST and other global health partners includes setting up, staffing country offices, and a step-by-step implementation approach. For the ESC countries, ASSIST’s task was to implement the improvement activity in multiple countries within a single year. Registering URC field offices within the countries and establishing project teams to locally support the operation was not feasible in the given timeframe. This constraint posed a significant challenge for logistics and implementation. To overcome the challenge, ASSIST undertook an innovative RMPP-MAPS which allowed simultaneous planning and execution of multiple startup processes in all four countries (instead of a linear, sequential fashion) and using real-time data or “feedback loops” to track progress. Given the common language, geographic proximity, and similar socio-economic status, URC was able to apply a universal approach. In documenting and evaluating this novel approach, URC has published observations and recommendations for similar rapid startup of donor-funded short-term technical assistance activities.

### Results of the start-up phase

The start-up activities in all four countries were conducted simultaneously except for the country visits, which were conducted sequentially by the start-up team visiting one country after the other. All four countries committed to taking on the work and to allocating the human resources needed for it. In the start-up discussions, differences between the resource availability in different countries became clear. For example, some countries had shortages in staff required to act as coaches, while others did not. These were factored into the workplan through arrangements with the Caribbean Regional Nurses Association (CRNA). By the end of the start-up phase all four countries embarked on implementing the workplan.

## Methods

### Evaluation design

A process evaluation design and a qualitative study methodology were employed to address the evaluation objectives ^9^. The evaluation was retrospective with primary data collected through in-depth interviews with persons or key informants (KI) involved in the startup and secondary data retrieved from the review of project documents. Documents reviewed included: (1) startup documents obtained from the ASSIST Project (e.g., meeting records, activity plans, timelines, presentations, trip reports, scoping survey, etc.), (2) online public resources (e.g., USAID Zika program overviews, press-releases, ESC country-specific information, literature related to Zika outbreak in the region, summary of relevant work by implementing partners, etc.), and (3) documents obtained from program managers (e.g., Maternal and Child Survival Program reports and documents). Primary and secondary data were triangulated as necessary to document and develop a cohesive picture of the activity. See *Extended Data* ^10^ for the documents reviewed.

### Identification and engagement of key informants

KIs were defined as individuals who had direct or indirect knowledge, experience, and/or involvement with any stage of the project development or startup activities. The USAID ASSIST Project provided a list of recommended KIs to the independent research consultant (B.A.) who conducted the interviews. The list included technical partners, country program planners, project coordinators, chairs and members of community and hospital nursing divisions, and maternal and child health committees. An initial email was sent to all prospective participants by the USAID ASSIST Project to introduce the consultant and inform the eligible KI about the evaluation. The consultant then contacted each KI via email, provided details about the evaluation purpose and rationale, and invited him or her to participate in an interview via Zoom (or in some cases, over the telephone). When necessary, the USAID ASSIST Project team facilitated contact with stakeholders to confirm their participation as a KI.

### In-depth individual interviews with key informants

A semi-structured interview guide ( *Extended data* ^10^) was structured to have sections covering key components of the startup identified from the preliminary discussions with ASSIST program officers. Initial discussions helped define the focus and objectives of the evaluation, precisely define the startup period and outline specific processes or activities that took place within the given timeframe. Key components included identifying startup activities in chronological order, elucidating views or understanding of the approach and methods employed, perceptions related to timeline and speed of the process, facilitating and hindering factors, and capturing lessons learned and future recommendations. Project documentation was used to describe the start up activities and identify additional topics for inclusion in the interview guide. Questions were adapted according to the type of respondent (national or regional partner, implementing partner, or leading STTA provider) and to fit the extent of participants’ involvement in the startup. All interviews were conducted in English and lasted approximately 45 minutes. The interviews were conducted by an independent, external research consultant (B.A.), who was not part of USAID ASSIST Project and was not involved in a project startup or implementation. The consultant holds a Ph.D. in Epidemiology and has expertise in public health research and program evaluation.

### Data analysis

All interviews were audio-recorded. Audio files were uploaded into password-protected computer files. Automated transcripts were then generated from interviews and transcribed using transcription software ( https://otter.ai). Transcripts were then manually checked and edited to ensure accuracy. The accuracy of transcription software was dependent on the respondent’s English accent (e.g., native English speakers in US vs. English of respondents from Caribbean countries), technical factors such as whether the interview was taken via phone, an online communication platform, or in-person, as well as recorded sound quality, presence of background noise, etc., and varied between 80 and 90 percent. Transcripts were not sent back to interviewees. Data were systematically grouped according to recurrent patterns and themes as related to central topics being evaluated (by B.A.), and codes were applied to identify segments of the interviews where these themes were discussed. The information obtained from the desk review was triangulated with the data retrieved from in-depth interviews to validate the information collected and more precisely map startup activities.

### Ethical statement

Informed consent was obtained from each KI prior to the interview via email (consent form available as *Extended data* ^10^). In addition, consent was obtained orally prior to recording each interview. Participants were informed of the voluntary nature of participation and the right to withdraw at any time or decline questions they did not wish to answer. Participants were also informed that their responses would be confidential (i.e., specific quotes or answers not linked to any specific respondent in the report). Interview audio, voice, and transcript records were stored securely in password-protected electronic files.

This evaluation was performed as part of the learning component of the USAID ASSIST Project and was not considered a research study subject to IRB review.

## Results

### Characteristics of evaluation respondents

Invitations to participate in the startup evaluation interviews were sent out to a total of 34 individuals, out of whom 28 (82.4%) were successfully interviewed. Interviews took place between January 29, 2019 and April 7, 2019. Interviewed participants comprised of key stakeholders involved in the startup, including technical assistance providers, implementers, and partners, based in USA and the Caribbean region. The distribution of respondents included: four USAID Washington staff; four URC staff working on the USAID ASSIST Project; one Jhpiego staff member who worked on the USAID Maternal and Child Survival Program (MCSP); three staff of the American Academy of Pediatrics (AAP); one representative of the Caribbean Regional Midwives Association (CRMA); one representative of USAID/Guyana (overseeing USAID’s regional activities in ESC); as well as stakeholders from the four technical assistance recipient countries. Respondent distribution is further illustrated in Table 1.

**Table 1.** Distribution of respondents.

Levels	Country	# invited	# interviewed
**Country-level**	**Antigua and Barbuda**	7	5
	**Dominica**	3	3
	**St. Kitts and Nevis**	6	2
	**St. Vincent and the Grenadines**	4	4
**Regional Level**	**Trinidad and Tobago: CRMA**	1	1
	**Guyana: USAID Regional Representative**	1	1
**Global Level**	**USA: USAID, ASSIST, MCSP, AAP**	12	12
**TOTAL**		34	28

Notes: *Six individuals did not participate, citing “family emergency”, “not being knowledgeable enough about startup activities”, and “no longer being attached to the program” as the reason for declining; three responded to invitation but were not interviewed due to scheduling conflicts or internet access/technical difficulties with Zoom*. *CRMA, Caribbean Regional Midwives Association; MCSP, USAID Maternal and Child Survival Program; AAP, American Academy of Pediatrics.*

### Stakeholders’ roles

**Figure 1.**
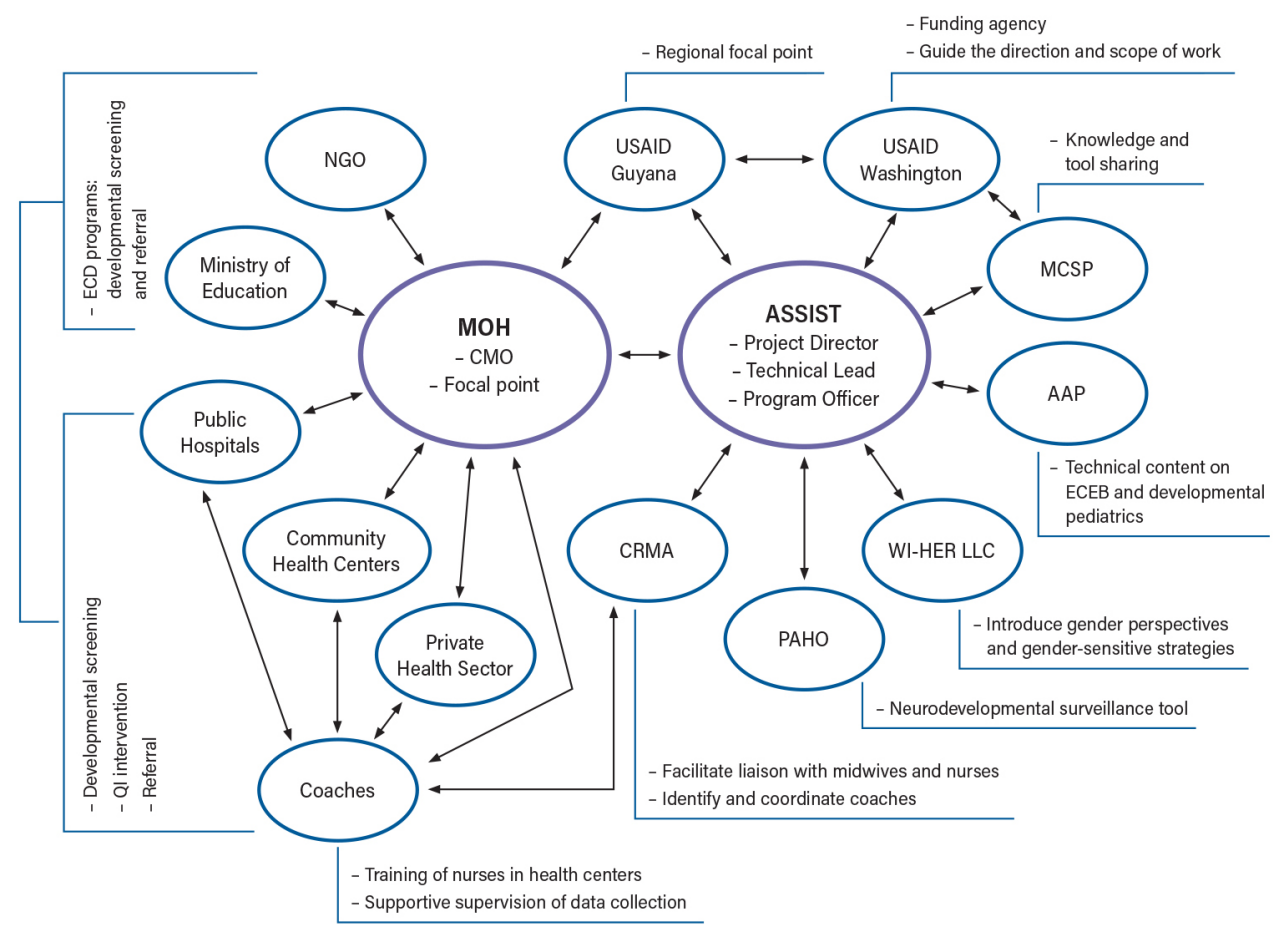
Map of stakeholders and communication/relationship linkages. Notes: The central part of the chart shows the country Ministry of Health as the principal technical assistance recipient and stakeholder and the USAID ASSIST Project as the leading provider and implementing partner of USAID. Key supporting implementing partners and other national stakeholders are arranged around the above two main stakeholders. Arrows reflect the relationship and communication flows between different stakeholders. Sidebars display the key roles each stakeholder played in the startup. USAID Guyana and CRMA are in the middle between the main stakeholders as regional liaisons and partners. MCSP, USAID Maternal and Child Survival Program; AAP, American Academy of Pediatrics; CRMA, Caribbean Regional Midwives Association; MOH, Ministry of Health; PAHO, Pan American Health Organization; CMO, Chief Medical Officer; WI-HER LLC, Women Influencing Health, Education, and Rule of Law; NGO, non-governmental organization; ECD, early childhood development; ECEB, essential care for every baby; QI, quality improvement.

Key stakeholders and their roles and responsibilities are shown in Figure 1. The ASSIST team in collaboration with country partners played a key role in the development of the overall scope, approach, activity plan, and implementation of the STTA. Jhpiego MCSP, a collaborating partner, worked closely with the ASSIST team to coordinate approaches and activities in the region and share lessons and experiences, as well as documents and training materials for adaptation and use in STTA. AAP, the project sub-awardee, was represented by Global Health Program Officers (management team) and pediatric consultants (technical team) and was engaged to bring in the subject matter expertise, specifically, to deliver training curricula on essential care for every baby (ECEB), and developmental pediatrics within the context of Zika. CRMA, the project sub-awardee, is a regional organization with members throughout the Caribbean region and was represented by an executive team. CRMA supported the selection and recruitment of quality improvement (QI) coaches. USAID’s Zika Program guided the overall direction and scope of the STTA.

### Startup activities and timeline

The startup consisted of multiple processes implemented in parallel in the four countries between May 11, 2018 (when the assignment was given to the USAID ASSIST Project) and September 3, 2018. In general, all activities fall under four main categories: (1) conception and initiation activities, (2) workplan and timeline development, (3) technical content development, and (4) operations. A detailed summary list of activities under each category is shown in Table 2.

**Table 2.** Summary list of startup activities.

Category		Activities
**Conception and initiation activities**	**Achieving buy-in**	Identify and contact key decision-makers in Ministry of Health to gauge interest and obtain initial support Determine country, regional stakeholders and US-based partners Initiate partnerships with stakeholders and sub-contractors Maintain communication, advocacy and engagement efforts to obtain full support and commitment of all concerned stakeholders Establish in-country activity teams (focal points, coaches, facility improvement teams, etc.) Start discussions with host country governments on sustainability and institutionalizing improvement activities
	**Developing focus and scope** **of TA**	Adapt and complete Scoping Visit Assessment Tool Conduct rapid baseline assessment of newborn and well-baby care systems with the focus on assessment of babies with suspected or confirmed congenital syndrome associated with Zika, and identify gaps, needs and priorities Plan and conduct country scoping visits with USAID global and regional representatives Develop the focus and scope of short-term technical assistance based on the scoping assessment and consultation with stakeholders Reach formal agreement with country stakeholders, USAID and technical partners on the scope of work
**Workplan & timeline** **development**		Develop detailed workplan with timeline and budget in close collaboration with USAID representatives, country partners and sub-contractors Submit workplan to USAID for review and approval
**Technical Content** **Development**	**Training curriculum &** **materials**	Quality improvement capacity building materials ZIKV-oriented early childhood development and essential care for every baby training materials Clinical capacity building materials and case studies based on the most current updates on Zika clinical care Neurodevelopmental surveillance and well-child care tools Pre/post knowledge assessment tools Capacity building materials for coaching support
	**Data** **collection**	Develop a set of indicators to track the progress of improvement activity Develop data collection reference guide Set up Improvement Indicator Database
**Operations**	**Logistics, administrative functions,** **communication, financial management**	Develop and approve sub-awards and agreements with partners Identify logistical needs (event organizers, information technology needs, supplies) Initiate multiple competitive procurement process including procurement of computers for health facilities, identifying workshop venues, accommodation, vendors for catering and transportation, etc. Develop/coordinate scheduling of activities with multiple stakeholders Find reproduction vendors and organize printing of training materials Finalize schedule, coordinate invitation and participation in workshop Arrange to award Continuous Medical Education credits to training participants through the countries' professional review boards Manage other administrative needs (documentation, reporting, etc.) Determine and streamline communication channels and principles between different stakeholders Determine accounting procedures, estimate cost and develop budget

**Figure 2.**
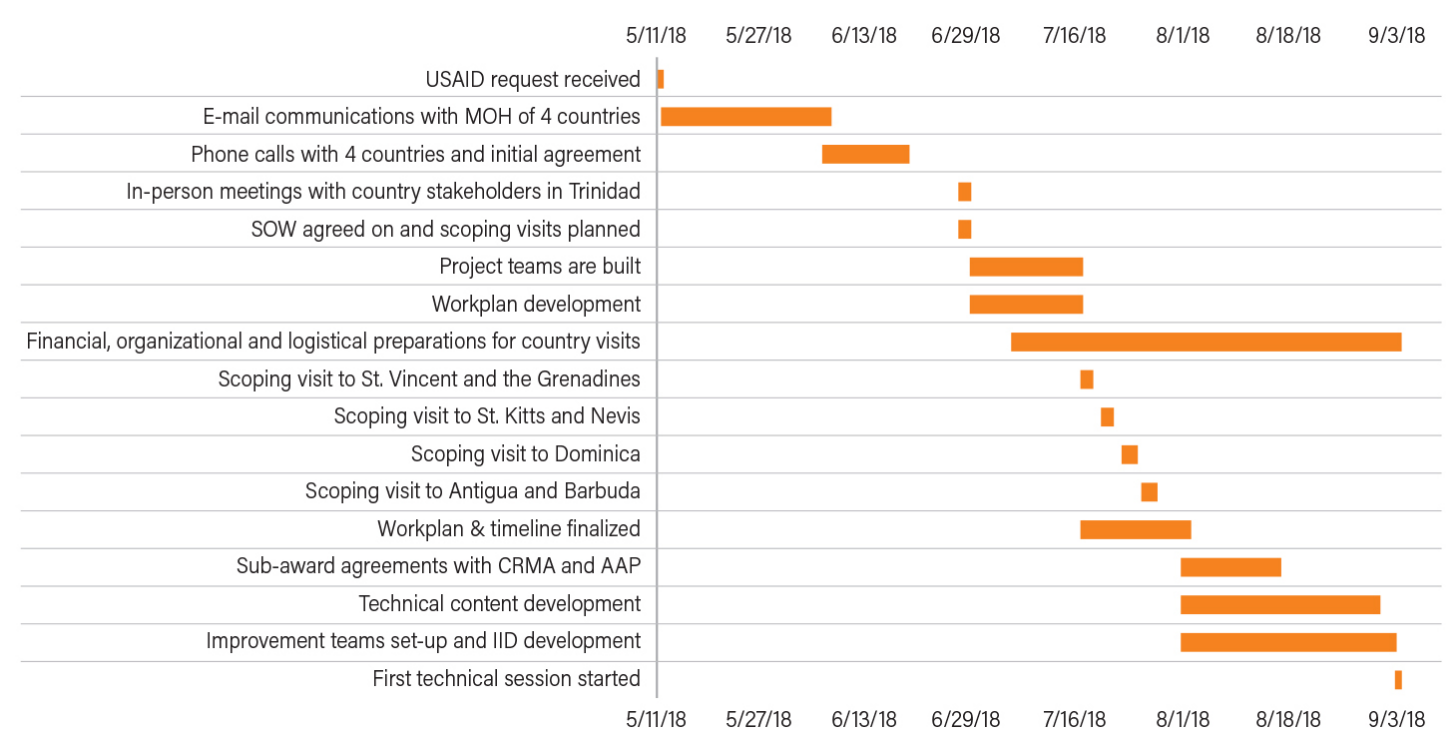
Timeline of key activities. AAP, American Academy of Pediatrics; CRMA, Caribbean Regional Midwives Association; MOH, Ministry of Health; SOW, Scope of Work; IID, improvement indicator database.

The timeline of key activities is shown in Figure 2. In less than four months, the USAID ASSIST Project was able to achieve buy-in from all countries, develop a work plan with approval from the USAID and the four Ministries of Health, develop technical content and capacity building materials for the first TA visit, initiate agreements with three sub-awardees and event planners, establish facility-based improvement teams in each country, determine indicators to track improvements in service delivery, and finalize financial, organizational and logistical arrangements for both the scoping and the first technical assistance visits.

### Key themes and issues identified from interviews

In this section, we describe and discuss some of the common themes and issues identified from interviews as related to key processes, challenges encountered, and any enabling and hindering factors.

***Achieving buy-in.*** Given the limited timeframe, it was critical to secure buy-in for USAID Zika expansion efforts from key stakeholders in the new ESC countries from the very beginning. Achieving buy-in involved: (1) describing the problem and providing a convincing rationale for why it needs to be addressed; (2) informing stakeholders of the availability of funds and technical expertise that can be leveraged by countries to co-develop and implement a solution; and (3) mapping out the project’s direct outputs/outcomes and extended benefits. Involving country stakeholders at each step was critical to achieving buy-in both at the decision making and implementation levels. However, there were several constraining factors when it came to engaging country stakeholders. First, ASSIST did not have a prior presence in these four countries. As explained by one respondent, there were "reservations" on the part of countries since they were not familiar with ASSIST or its work. Second, by the time USAID approached countries, the Zika epidemic and the heightened urgency around Zika was essentially over. Finally, the countries faced competing priorities such as dealing with the aftermath of devastating hurricanes that ravaged the region in 2017. To address the countries’ needs, the activity focused on detection and support of babies potentially affected by Zika through strengthening essential newborn and well-baby care systems and early childhood development programs.

According to USAID and ASSIST respondents, initially, there were delays in establishing regular communications with the countries. Different “levels of engagement” were noted, with some country partners being more responsive compared to others. These challenges were mitigated through ASSIST’s continuous engagement with the USAID representative from the regional office, who facilitated direct contact with country stakeholders via email and phone. ASSIST also coordinated face-to-face meetings at a regional conference in Trinidad and during country scoping visits to ensure full commitment from country partners.

From the countries’ perspectives, the demonstrated experience of the USAID ASSIST Project and the proposal laid out during the initial discussions were noted as influential in deciding to get on board with the activity. As one country senior Ministry of Health (MOH) officer described:

*“First of all, I found (USAID ASSIST) extremely knowledgeable. They have a wealth of experience and have done extensive work in quality all over the world. So, that for us was really important to know. Second, I think that they were quite sensitive to the specificities of all cultural nuances and …were quite responsive to suggestions we made and how to go forward. It was not just cordial… after a couple of weeks, we were talking like we've known each other for years. So, I think, that speaks to the commitment both parties had to this as well as the experience that USAID ASSIST has”*.
                                                                                                                                                                                                                                                              
*(Senior MOH officer)*

Additional contributing factor for accepting the USAID Zika STTA was the recognition of the need to have ongoing Zika surveillance and up-to-date national policy documents and guidelines for developmental surveillance, screening, referral, and support of children potentially affected by Zika. Country pediatricians have noted that the existing policy documents and guidelines on Zika were developed during the height of the epidemic and did not reflect the new evidence. For example, in Antigua and Barbuda, the guidelines for infants exposed in utero to ZIKV were developed in 2016 with technical assistance from Pan American Health Organization (PAHO). This was useful for clinical providers in responding to the outbreak at the time; however, the knowledge and evidence about Zika has since expanded. Additionally, terminology, including the definition of congenital syndrome associated with Zika (CSaZ), was not reflected. Therefore, the USAID Zika technical assistance was viewed by leading pediatricians in these countries as a “fantastic” opportunity to update relevant policy documents with the help of ASSIST and AAP.

*"First of all, we recognize what we should follow in terms of surveillance, that we need to ensure we have the same case definition, that we have national policies, and we recognize that there is a limitation in terms of human resources. We also recognize that there needs to be someone external, who is looking into the weaknesses within the system, and that's one of the reasons that we were happy to have technical assistance. This is how we could improve because we're good on paper, we have many things in place, but the system needed to be improved and (made) more robust."*
                                                                                                                                                                                                                                                                 (Country team member)

***Defining STTA focus, scope and approach.*** Interviews with USAID respondents revealed that USAID designed a response framework around the four major lines of effort, each addressing a specific factor in preventing and controlling the spread of the Zika epidemic. The four lines of effort were: (1) vector control; (2) social and behavior change, communication, and community engagement; (3) service delivery; and (4) research, development, and innovation. Depending on the stage of the epidemic and the needs of the countries, implementing partners were guided by USAID to focus their efforts on one or more particular lines of effort to strategically align response activities amongst the various partners. Since ASSIST started its efforts in ESC countries, nearly two to three years after the outbreak had peaked, the country priorities shifted towards improving service delivery around identification of cases of CSaZ and other developmental malformations among infants and children exposed to ZIKV in utero and strengthening newborn and well-baby care systems to address other similar emergencies.

*“The disease epidemiology shifted from when the first outbreak started, …trying to minimize another outbreak …to care and support for those who have been affected. Now, we are …trying to answer the question about the transition from emergency response to long-term development.”*
                                                                                                                                                                                                                                                              (USAID Zika team member)

**Figure 3.**
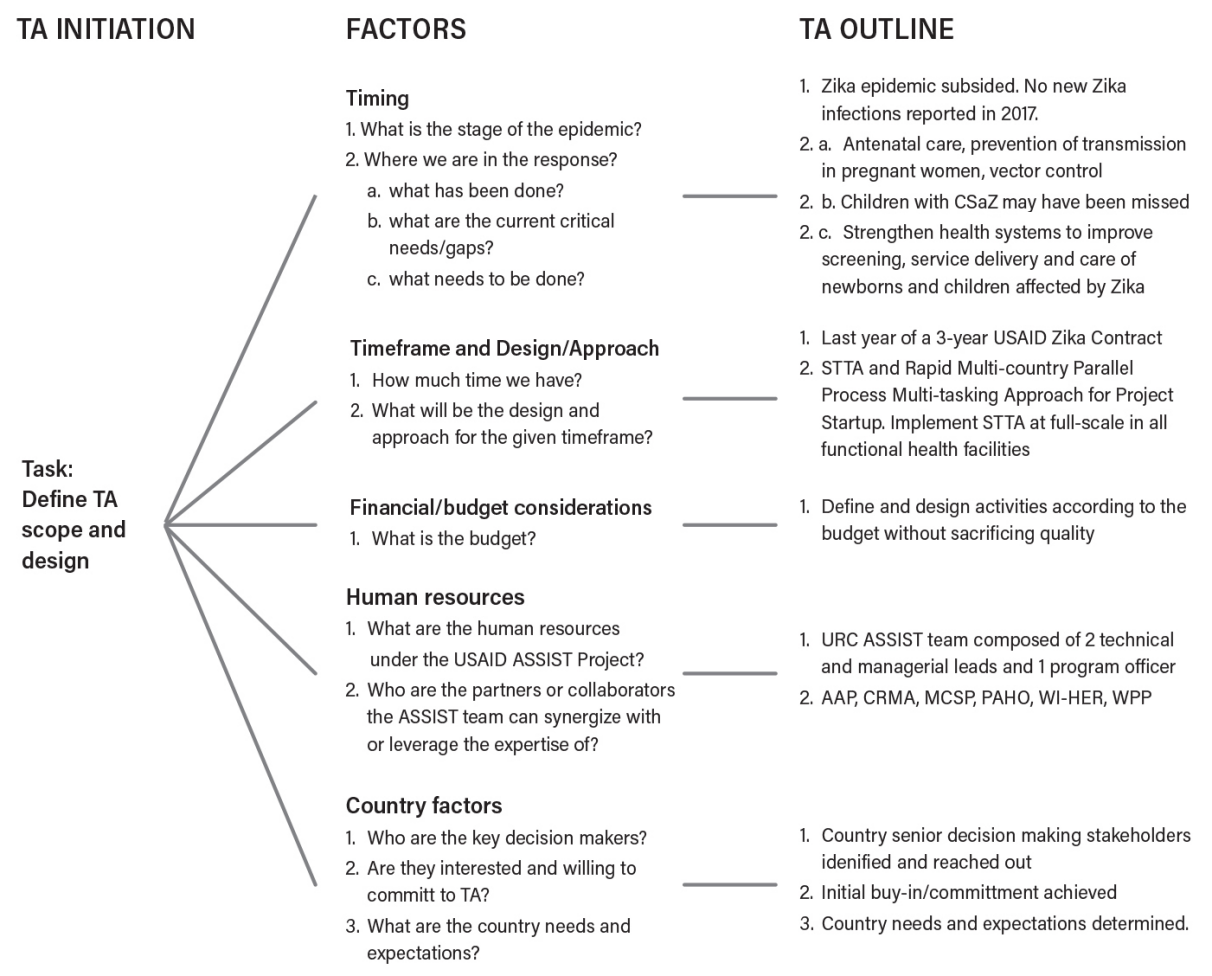
Key determining factors for the STTA focus, scope and approach. TA, technical assistance; CSaZ, congenital syndrome associated with Zika; STTA, short-term technical assistance; MCSP, USAID Maternal and Child Survival Program; AAP, American Academy of Pediatrics; CRMA, Caribbean Regional Midwives Association; PAHO, Pan American Health Organization; WI-HER LLC, Women Influencing Health, Education, and Rule of Law; URC, University Research Co., LLC; WPP, World Pediatric Project.

Therefore, the focus at this stage of the response was to improve and strengthen national health systems to enhance national capacities to address public health emergencies. Based on responses from USAID and country participants, the following factors have played a role in shaping the focus, scope and approach of the STTA: (1) timing or the current phase of the Zika epidemic; (2) timeframe of the USAID Zika response contract; (3) financial and human resources; and (4) country-level factors ( Figure 3).

All MOH focal persons and relevant decision-makers were engaged in the process of co-developing the work plan and finalizing the technical and geographic scope of the work.

Technical partners with specific areas of expertise were contracted after the scope and the types of technical expertise that would be needed had been defined. However, some partners wished they were involved in initial discussions. According to technical partners, earlier engagement would have helped define expectations, roles, and responsibilities clearly from the beginning while giving the partners time to plan their part of work according to the country contexts. The time left after achieving country buy-ins and initial discussions with countries to define TA focus and scope was less than a month.

***Perspectives on the RMPP-MAPS***. The approach was based on the ASSIST’s “model for improvement” ^11^ and “adaptive management” ^12^ strategies. These strategies emphasize identification of gaps and testing of local solutions to problems in real time, adjusting plans throughout the process, and implementing the results of regular monitoring of improvement interventions. Among the advantages of a rapid approach, respondents noted faster implementation, early achievement of results, expedited scaling and delivery of patient-favorable outcomes into the health services, and the efficient use of resources. Disadvantages included a possible loss of specificity and the potential for missing subtle details in the design during the early stages of response. Only a few respondents raised concerns about the possibility of diminished quality of response. Respondents emphasized that a continuous feedback mechanism and real-time adaptive management and learning strategies helped to address quality or other concerns promptly. While constant feedback and inputs from all partners were actively sought and incorporated at each step, the limited time frame made it impossible to wait until all components and specific details were in place or agreed upon by all stakeholders. Rather, the strategy adopted by ASSIST was to proceed with all necessary startup activities simultaneously to avoid delays while refining and/or making adjustments along the way as needed.

*The only way to improve care is to change what is happening at facilities. So, our goal was to start facility-level improvement activities at the earliest point as possible. Knowing from previous experiences that planning is most effective when it is informed by implementation, we tried to start as fast as possible and use robust continuous feedback loops at every level to learn and adapt.*”
                                                                                                                                                                                                                                                              (ASSIST team member)

***Scale up and engagement of local stakeholders.*** The small population and geographic size of the four countries facilitated the full scale of the improvement activity from the start. Except for the few community-based clinics in Barbuda and Dominica that were damaged by hurricanes, all facilities were included in the improvement intervention. To promote early adoption and institutionalization of changes and cross-sectoral collaboration ASSIST engaged health workers at all levels of the health system and encouraged the involvement of early childhood developmental programs under the Ministry of Education, and other community-based organizations (e.g., Roving Caregivers Program in Dominica). Both scaling up and institutionalization are essential components of ASSIST’s health service improvement model. These features of the STTA were noted by many country respondents as unique when compared to other technical assistance projects they had experience within their countries. According to country representatives, often in such projects, information "stays at the top" while "people at the bottom" are left poorly informed and tasks are "thrown" at them. Many pointed out that frontline and mid-level health workers (and, in some cases, community health aides and nursing assistants) had benefited greatly from training on clinical care of Zika-affected newborns and children. The science of QI empowered health workers to improve processes within the newborn and well-baby care system and equipped them with the skills and knowledge to apply improvement principles to other priority clinical-content areas.

Ensuring the participation of all stakeholders was not without challenges. The health systems in three of the four countries (except St. Vincent and the Grenadines) were suffering from severe shortages of health care workers, particularly nurses. Many nurses and other health care workers had migrated out to other countries following recent hurricanes, exacerbating pre-existing shortages. Hence, some health facilities were concerned additional project-related responsibilities would overwhelm staff members. Due to this shortage of local health workers and heavy workload, country partners and ASSIST used retired nurse midwives who were already familiar with the health system. St. Vincent and the Grenadines was the only country that did not request external help to provide on-site coaching support to health facilities. According to a MOH officer, there was no shortage of nurses in the country. St. Vincent and the Grenadines relied on the existing nurse supervisors to take on coaching responsibilities. St. Vincent and the Grenadines did, however, experience the same challenge as the other countries when it came to "pulling the staff" for a week-long technical session while ensuring no interruption in health services.

Country teams and facilities had to shuffle various schedules and calendars around clinic days or public holidays to prevent disruption of health services and accommodate project activities.

*“Well, the most stressful part of it was getting the persons together…, that was kind of a tedious job getting into different entities. The time that we had in order to prepare for that first visit and the categories of individuals that they were requesting to meet with, the timeframe was a bit short in order to get all those persons together. Persons need prior notice in order to be released. We weren't giving them that prior notice. We are basically giving them “a meeting is tomorrow and I need X number of staff”. They don't have that time to prepare…”*
                                                                                                                                                                                                                                                              (Country team member)

Country team leaders noted that promoting a better understanding of the improvement activity, the benefits of adopting changes, and support from ASSIST helped to overcome the initial resistance. As one country focal point put it, they had to “sell the idea” to staff. A dedicated leadership team and local champions were crucial in creating a “positive competitive environment” to motivate increased participation and involvement. The result was increased interest and demand for participation in technical learning sessions observed from the second TA visit, with more health providers attending the workshops than initially planned.

***Technical content development.*** Technical content development focused on two major areas: (1) the science of QI led by the ASSIST team; and ECEB training with a focus on ZIKV led by AAP. One of the challenges identified by respondents was the lack of technical experts with expertise in both ECEB and ZIKV. According to AAP, the pool of experts trained in ECEB was limited and finding available experts to work on developing the curriculum on “a very short turnaround time” presented significant challenges. Nevertheless, the AAP team was instrumental in “pulling together” their existing materials and information resources, as well as a team of consultants to develop high-level technical tools. In mitigating short timeframe challenges, AAP and other implementing partners emphasized the importance of early involvement of technical partners in the rapid startup. Additionally, providing technical consultants with as much background or context data on local health systems and related issues in advance, was highlighted as an essential best practice.

***Operations.*** Operations included functions related to defining administrative support (documentation, reporting, contracting, etc.), financial management (budgeting, accounting, payments, etc.), streamlining communications between multiple stakeholders, and logistical needs (procurement/supply, event organization, etc.). Given the lack of field offices and URC staff within the countries, administrative and financial management was centralized from ASSIST headquarters in Chevy Chase, MD, USA with country-specific adjustments made where necessary.

Logistical activities were outsourced to external organizations. The main challenge was identifying reliable event planning or logistics services on the ground who have intimate knowledge of local markets, suppliers, distributors, or experience with procurement management and competitive purchasing processes. For example, no reputable event planning organizations meeting the minimum required qualifications were identified in St. Vincent and the Grenadines. A unique logistical challenge for St. Vincent and the Grenadines were the delays arranging transportation for the "nurses from the Grenadines to come to the mainland" (some of the Grenadines’ nine islands are small and remote). Therefore, ASSIST contracted two independent consultants to take on the role of event planners. The ASSIST team worked with MOH focal points in all four countries to help identify and provide recommendations on potential vendors and co-implement activity to help "build local ownership and capacity."

Financial management followed USAID accounting procedures and standards according to the terms of the ASSIST cooperative agreement between USAID and URC. Planning and budgeting took place at the same time as workplan, staffing, and operational needs were being defined. Difficulties were reported in prompt costing of local activities.

Figure 1 illustrates the flow of communication between all stakeholders and partners. The ASSIST team and the focal points from the country teams were the two parties in primary contact through whom all messages from other partners were communicated. This was established to ensure consistency of messages across multiple partners. According to ASSIST, the sub-awardees were not in direct communication with country stakeholders during the early start-up phase to facilitate focused conversations about buy-in or the technical scope. However, relevant updates and decisions were shared in a timely manner. Communication was open and regular, informing all relevant stakeholders, including local stakeholders and partners. There were also regular conference calls involving stakeholders from all islands. Emails to relevant stakeholders and partners were answered within 24 hours, and urgent issues were handled through telephone, Zoom calls, and WhatsApp.

***The speed or pace of the startup***. Participants reported that given the substantial amount of work in preparation for the first TA session in early September and very short timeframe, the startup had to be rapid and fast-paced, which had both advantages and disadvantages. The rapid startup galvanized stakeholders to brainstorm and make decisions quickly. However, the rapid speed may not have allowed enough time for detailed or thorough follow-up of some tasks (e.g., not all materials were printed out in time for the first technical session). When asked about how the pace of the startup compared to other projects, most respondents indicated that they are not aware of any other projects of the scale, depth, or the speed and pace that occurred with the USAID ASSIST Project. Both ASSIST team members and partners reported exerting a great level of effort to manage multiple tasks successfully and efficiently at the same time within a short timeframe.

## Lessons learned and recommendations

This evaluation revealed that a rapid startup of complex service delivery technical assistance with activities in multiple countries without field offices was feasible. Although there were several unexpected implementation bottlenecks, by the end of the startup there was a collective realization among project teams and partners that what seemed at first a “mission impossible” was, in fact, possible, and had been accomplished. In addition, the evaluation proved that in the context of a short timeframe and urgent nature of the task, defining essential startup steps and processes early in the course of activity while at the same time identifying and accounting for specific contextual challenges is critical to avoid delays and facilitate smooth execution.

Additional lessons gleaned from respondents’ comments are summarized and listed below. While some of these lessons are not unique to the experience of the USAID ASSIST Project, they were distinctly reinforced by the course of startup and proved to be valuable for future startups.

*At technical assistance/implementing partner level:*

- To achieve buy-in:
  ◦ Maintain regular dialogue with senior country-level decision-makers to achieve prompt buy-in
- When developing response/designing TA:
  ◦ Involve implementing partners and collaborators early
  ◦ Establish efficient but flexible communication principles
- When setting up operations:
  ◦ Learn as much as possible about host country context and determine optimal operational mechanisms, including:
    ■ Identify knowledgeable individuals and/or entities locally to take on or share logistical responsibilities
    ■ Determine context-appropriate contracting and financial management strategies

*At the country/beneficiary level:*

- When co-developing and co-implementing TA:
  ◦ Conduct rapid assessment of the health system and service delivery needs jointly with key national stakeholders and subsequently with implementing partners
  ◦ Co-develop interventions with a focus on sustaining the impact at scale, tailoring TA to country context, capacity development needs, and achieving local ownership of the activity
- When implementing TA:
  ◦ Ensure activities are naturally integrated within the existing system, structures, and functions and not run or perceived by staff as a “parallel” task. Create a positive environment among health workers and avoid or mitigate resistance to change
  ◦ Establish routine communication with key country stakeholders, partners, and donors to update them about progress, respond to their needs, promptly communicate challenges, and seek joint solutions
  ◦ Identify and mitigate implementation barriers
    ■ Gather intelligence on logistical challenges from key local stakeholders
    ■ Identify and engage reliable or experienced local vendors early (before initial scoping visit) to support logistics of field activities, avoid delays, and ensure rapid start-up of TA activities

Both TA providers and beneficiary countries should aim to find an optimal balance between country needs and/or expectations and what technical partners and donors can offer, and to identify and acknowledge various limitations (programmatic content, financial, human resources, etc.) in advance. This will facilitate successful collaboration and ensure the achievement of project goals and outcomes.

Finally, the presence of committed leadership, dedicated project teams, and local champions with the ability to collaborate on project strategy and adaptation was repeatedly emphasized as a critical process. The latter point consistently comes up as an essential factor in all projects ASSIST has implemented to date globally ^13, 14^.

### Limitations

Recall bias is a potential study limitation as interviews took place four to six months after the startup in the middle of the implementation phase. Participants sometimes had difficulties recalling details related to activities, dates, and sequences of events, and could not always distinguish startup from implementation activities. There is also a possibility for social desirability bias in response to sensitive questions (e.g., what the participant did not like about startup). There is also chance for respondent or KI selection and inclusion process. However, the risk is believed to be minimal as all individuals involved at any stage of the startup were identified as potential KIs, contacted and invited to participate in the study with response rate exceeding 80 percent. 

## Conclusions

In less than four months, the USAID ASSIST Project developed and carried out an innovative RMPP-MAPS to lay the groundwork for the implementation of a complex STTA activity in the four Caribbean countries. The STTA was implemented in full scale in all four countries and engaged health workers, educators, and caregivers at all levels of the newborn and well-baby service delivery system, promoting early institutionalization and long-term sustainability of the evidence-based interventions.

Challenges encountered in the startup stemmed from the tight timeline, the lack of in-country offices, and the shortage of healthcare staff in the ESC countries. Despite these challenges, the RMMP-MAPS model for startup facilitated a rapid launch of the implementation phase of the STTA. Dedicated project teams, country leadership, local champions, and a strong sense of partnership and country ownership were identified as critical factors in facilitating a successful startup.

The RMPP-MAPS is a feasible and resource-efficient model to consider by implementers, donors, and low-and middle-income countries facing temporal and financial constraints when responding to public health emergencies.

## Data availability

### Underlying data

In order to encourage free and open sharing of opinions and perceptions, we assured stakeholders that their responses would be confidential. Since interview questions were shaped around a participant’s specific role and involvement level in the startup, their responses were correspondingly specific to their particular experiences and views. Many organizations were represented by no more than 1–2 respondents. Hence, interview files (transcripts and audio recordings) will contain potentially identifying information and thus, cannot be made openly available. Those interested in accessing this data may contact the corresponding author, at rashadmassoud@outlook.com. Requests will be considered on a case-by-case basis and must include contact information, type of data requested, and justification (e.g., proposal) for how the data will be used and for what purposes. Any information that could potentially identify respondents or organizations will be removed from the data shared.

### Extended data

Open Science Framework: Zika Startup Evaluation Manuscript Extended Data, https://doi.org/10.17605/OSF.IO/B7QGS ^10^

This project contains the following extended data:

- Short-Term Technical Assistance startup review documents
- Semi-structured interview guides
- Consent form

Data are available under the terms of the Creative Commons Zero "No rights reserved" data waiver (CC0 1.0 Public domain dedication).
