# Supplementary material for: Evaluation of the rapid, multi-country, parallel process, multi-tasking approach to startup of short-term technical assistance to improve service delivery in newborn and child health in the context of USAID’s Zika response in four Eastern and Southern Caribbean countries
Source: F1000Res. 2022 Apr 4;9:251. Originally published 2020 Apr 9. [Version 3] doi: 10.12688/f1000research.22814.3 (PMC8980677; doi:10.12688/f1000research.22814.3)
Supplement: Peer review history

# Peer review: Reviewer response for version 3

**DOI:** 10.5256/f1000research.122715.r129691

**Published:** 2022-04-04

**Authors:** Lisa Hirschhorn

**Affiliations:** 1 Feinberg School of Medicine, Northwestern University, Chicago, IL, USA

The authors have responded to the comments and the article is approved for indexing from my perspective.

Is the work clearly and accurately presented and does it cite the current literature?

Partly

If applicable, is the statistical analysis and its interpretation appropriate?

Yes

Are all the source data underlying the results available to ensure full reproducibility?

Partly

Is the study design appropriate and is the work technically sound?

Partly

Are the conclusions drawn adequately supported by the results?

Partly

Are sufficient details of methods and analysis provided to allow replication by others?

Partly

Reviewer Expertise:

Implementation science, QI, MNCH.

I confirm that I have read this submission and believe that I have an appropriate level of expertise to confirm that it is of an acceptable scientific standard.

# Peer review: Reviewer response for version 2

**DOI:** 10.5256/f1000research.119931.r121276

**Published:** 2022-02-16

**Authors:** Lisa Hirschhorn

**Affiliations:** 1 Feinberg School of Medicine, Northwestern University, Chicago, IL, USA

The authors have provided in-depth responses to both reviewers' comments. However, while some of these responses are reflected in the revised manuscript, others remain in the reviewer responses. Matching these carefully crafted responses into edits would strengthen the paper and fully address the reviews' questions. For example, the qualitative analysis methods, the question about aligning the scope of the paper ("An evaluation was conducted to document the STTA startup activities, to identify enabling and constraining factors, and to capture lessons learned."), while other parts talk about the full implementation (albeit with less details than start-up) and sustainability. A specific focus of the paper at the end of the introduction could help orient the reader. Clarity on the goals and, if included, linking implementation to the start-up if the data allows, detailing a bit more about the TA. Addressing all the comments through edits (or noting if not and why) will significantly strengthen this important paper.

Is the work clearly and accurately presented and does it cite the current literature?

Partly

If applicable, is the statistical analysis and its interpretation appropriate?

Yes

Are all the source data underlying the results available to ensure full reproducibility?

Partly

Is the study design appropriate and is the work technically sound?

Partly

Are the conclusions drawn adequately supported by the results?

Partly

Are sufficient details of methods and analysis provided to allow replication by others?

Partly

Reviewer Expertise:

Implementation science, QI, MNCH.

I confirm that I have read this submission and believe that I have an appropriate level of expertise to confirm that it is of an acceptable scientific standard, however I have significant reservations, as outlined above.

# Response

**Published:** 2022-03-20

**Authors:** M. Rashad Massoud

**Affiliations:** Department of Public Health, URC, USA

Dear Lisa,

 The evaluation was conducted on the start-up period of the project from the initial receipt of the assignment to the point at which technical activities were started. It is specifically about how we rapidly started up the project in four countries simultaneously - working with the four Ministries of health; connecting with other stakeholders in each country as appropriate; forming improvement teams in multiple facilities in each country; and creating the supervisory, learning and improvement networks between them. This study is not an evaluation of the project itself. The project is referred to only in the context of giving the reader a better understanding of the nature of what was started and why it was important to do so rapidly. I hope this addresses your question. Thank you.

 Kind regards, Rashad

# Peer review: Reviewer response for version 2

**DOI:** 10.5256/f1000research.119931.r121275

**Published:** 2022-02-10

**Authors:** Manoja Kumar Das

**Affiliations:** 1 The INCLEN Trust International, New Delhi, Delhi, India

The previous comments have been addressed and if the remaining comments below are addressed, the manuscript may be approved.

- The Methods section should include that 'inductive or thematic analysis' was adopted for the analysis and interpretation.
- As the data were analyzed by one person/author, it may be mentioned in the limitation section.

Is the work clearly and accurately presented and does it cite the current literature?

Yes

If applicable, is the statistical analysis and its interpretation appropriate?

Yes

Are all the source data underlying the results available to ensure full reproducibility?

Yes

Is the study design appropriate and is the work technically sound?

Yes

Are the conclusions drawn adequately supported by the results?

Partly

Are sufficient details of methods and analysis provided to allow replication by others?

Partly

Reviewer Expertise:

Public health

I confirm that I have read this submission and believe that I have an appropriate level of expertise to confirm that it is of an acceptable scientific standard.

# Peer review: Reviewer response for version 1

**DOI:** 10.5256/f1000research.25189.r99249

**Published:** 2021-11-22

**Authors:** Manoja Kumar Das

**Affiliations:** 1 The INCLEN Trust International, New Delhi, Delhi, India

This report presents the process and experience of the technical assistance to the four LAC countries for addressing the Zika challenge. The documentation and dissemination are important for lessons and potential application in the future.

 The manuscript/article can be improved by considering the following points:

**1. Data collection and analysis**

 1.1. How were the KI IDI guides drafted/developed? It should be mentioned. 

 1.2. Which method was used for qualitative data analysis? It should be mentioned. 

 1.3. Was the data analysis done by only one person (B.A.)? Usually, qualitative analysis is done by more than one person. 

 1.4. What was the degree of error with the use of the transcription software? 

**2. Results**

 2.1. How did the status of preparedness, responses, perceptions, practices, and response components vary across these countries? These should have been mentioned. 

 2.2. Triangulation with the record review/desk review has been mentioned in methods. The observations have not been mentioned clearly in the results section.

 2.3. Some narration about the results of the start-up activities should be given - whether the countries carried on the activities as planned or not.

 2.4. The conclusion mentions the TA as a resource-efficient model. Some information about the resource-efficiency should be mentioned.

Is the work clearly and accurately presented and does it cite the current literature?

Yes

If applicable, is the statistical analysis and its interpretation appropriate?

Yes

Are all the source data underlying the results available to ensure full reproducibility?

Yes

Is the study design appropriate and is the work technically sound?

Yes

Are the conclusions drawn adequately supported by the results?

Partly

Are sufficient details of methods and analysis provided to allow replication by others?

Partly

Reviewer Expertise:

Public health

I confirm that I have read this submission and believe that I have an appropriate level of expertise to confirm that it is of an acceptable scientific standard, however I have significant reservations, as outlined above.

# Response

**Published:** 2021-12-12

**Authors:** M. Rashad Massoud

**Affiliations:** Department of Public Health, URC, USA

Response to Reviewer #2:

 The manuscript/article can be improved by considering the following points:

**1. Data collection and analysis**

 1.1. How were the KI IDI guides drafted/developed? It should be mentioned. 

**Author response:** *To draft KI interview guides initial list of topics of interest to be evaluated around the startup components was developed based on preliminary discussions with ASSIST program officers. These initial conversations helped define the focus and objectives of the evaluation, develop a comprehensive list of stakeholders and key informants involved at any stage of the startup, precisely define the startup period and outline specific processes or activities that took place within the given timeframe.*

*Additionally, project documentation, including meeting minutes, presentations, trip reports, work plans, USAID and partners' program documents, etc., were reviewed to more precisely construct activities and identify additional topics of interest for inclusion in the interview guide.*

*The interview guide was structured to have sections built around identified topics and startup components with specific questions and probes drafted to facilitate getting answers for evaluation objectives. Prepared draft tools were reviewed by ASSIST, and received feedback was used to finalize the tools.*

*We have described the above development process in the manuscript, in brief, to keep the length of the manuscript within the recommended guidelines.*

 1.2. Which method was used for qualitative data analysis? It should be mentioned. 

**Author response:** *The number of respondents was not large, under 30, but respondents varied widely by which stakeholders or countries they represented. Additionally, interviews were conducted in a free-flow conversation style which allowed a diverse range and at the same time often unique answers or specific issues raised by respondents. Therefore, to best capture the range, the grouping or coding of emerging common and unique response themes was done manually (i.e., no automated qualitative analysis software or tools were used).*

 1.3. Was the data analysis done by only one person (B.A.)? Usually, qualitative analysis is done by more than one person. 

**Author response:** *Data analysis was done by B.A., who was not involved in the project and contracted to perform an independent evaluation of the activity and avoid potential bias if the evaluation was to be done by ASSIST people themselves. The evaluation was performed as part of the learning process of the project. It was not considered a research subject to IRB review, which would have, perhaps, required more rigorous data analysis or verification strategies.*

*However, all collected data (audio records, transcripts of interviews) were submitted to ASSIST and independently verified by supervising program officer who was not involved in the startup activities for completeness, data integrity, and record-keeping purposes according to program rules.*

 1.4. What was the degree of error with the use of the transcription software?

**Author response:** *The accuracy of the transcription software varied depending on the respondent’s English speaking style and accent (native English speakers in the US vs. English of respondents from Caribbean countries). Also, technology factors such as whether the interview was via phone, or Zoom platform, in-person, recorded sound quality, presence of background noise, etc., played a role. On average, the accuracy was between 80 and over 90%.*    

**2. Results**

 2.1. How did the status of preparedness, responses, perceptions, practices, and response components vary across these countries? These should have been mentioned. 

**Author response:** *The four countries were similar in terms of economic development level or health infrastructure state. As relevant to the startup, most differences were observed in the levels of availability or the shortage of local health care providers, particularly nurses; availability of qualified local vendors for outsourcing operational and logistical tasks; as well as relatively poor technological infrastructure in facilities, in terms of availability of computers, internet connectivity, even electricity in some cases. The latter was partly due to the differing severity of damages caused by hurricanes that passed through the region the prior year. Hence, priorities or promptness in responsiveness varied somewhat between countries but consistent follow-up and identifying bottlenecks in a timely manner helped the team address the challenges and needs without significant delay and allow simultaneous initiation of the technical phase of the TA. *  

 2.2. Triangulation with the record review/desk review has been mentioned in methods. The observations have not been mentioned clearly in the results section.

**Author response:** *Because KI typically were few in numbers representing different stakeholders and each played a unique role in the project, no one informant could provide a comprehensive picture “from the start to the end.”*

*Therefore, triangulation or tracing and comparing data obtained from multiple sources (preliminary discussions, in-depth interviews, project documents, etc.) was done to corroborate information, construct a cohesive and accurate picture of the startup, deduce the timeline and sequence of events, or elucidate enabling and limiting factors in order to reach evaluation objectives.*

 2.3. Some narration about the results of the start-up activities should be given - whether the countries carried on the activities as planned or not.

**Author response:** *The start-up activities were implemented in all four countries as described.*

 2.4. The conclusion mentions the TA as a resource-efficient model. Some information about the resource-efficiency should be mentioned.

**Author response:** *No specific cost analysis was done as part of this evaluation. The conclusion was based on the previous experience of ASSIST program leaders and TA implementers, other stakeholders, who had decades of experience in implementing similar health systems strengthening TA in multiple countries in different regions. Similar TA under normal circumstances typically involved setting up country offices, hiring local staff, external and internal consultants with startup activities taking much longer, often over a year incurring significantly higher costs before TA is ready to launch.*

# Response

**Published:** 2022-01-04

**Authors:** M. Rashad Massoud

**Affiliations:** Department of Public Health, URC, USA

The manuscript/article can be improved by considering the following points:

**1. Data collection and analysis**

 1.1. How were the KI IDI guides drafted/developed? It should be mentioned. 

- **Response**: To draft KI interview guides, an initial list of topics of interest to be evaluated around the startup components was developed based on preliminary discussions with ASSIST program officers. These initial conversations helped define the focus and objectives of the evaluation, develop a comprehensive list of stakeholders and key informants involved at any stage of the startup, precisely define the startup period and outline specific processes or activities that took place within the given timeframe.
  Additionally, project documentation, including meeting minutes, presentations, trip reports, work plans, USAID and partners' program documents, etc., were reviewed to more precisely construct activities and identify additional topics of interest for inclusion in the interview guide.
  The interview guide was structured to have sections built around identified topics and startup components with specific questions and probes drafted to facilitate getting answers for evaluation objectives. Prepared draft tools were reviewed by ASSIST, and received feedback was used to finalize the tools.
  We added the above additional details to the revised version.

**1.2. Which method was used for qualitative data analysis? It should be mentioned. **

- **Response**: The number of respondents was not large, under 30, but respondents varied widely by which stakeholders or countries they represented. Additionally, interviews were conducted in a free-flow conversation style which allowed a diverse range and at the same time often unique answers or specific issues raised by respondents. Therefore, to best capture the range, the grouping or coding of emerging common and unique response themes was done manually (i.e., no automated qualitative analysis software or tools were used).
**  **

**1.3. Was the data analysis done by only one person (B.A.)? Usually, qualitative analysis is done by more than one person. **

- **Response**: Data analysis was done by B.A., who was not involved in the project and contracted to perform an independent evaluation of the activity and avoid potential bias if the evaluation was to be done by ASSIST people themselves. The evaluation was performed as part of the learning process of the project. It was not considered a research subject to IRB review which would have, perhaps, required more rigorous data analysis or verification strategies.
  However, all collected data (audio records, transcripts of interviews) were submitted to ASSIST and independently verified by supervising program officer who was not involved in the startup activities for completeness, data integrity, and record-keeping purposes according to program rules. 

**1.4. What was the degree of error with the use of the transcription software?**

- **Response**: The accuracy of the transcription software varied depending on the respondent’s English-speaking style and accent (native English speakers in the US vs. English of respondents from Caribbean countries). Also, technology factors, such as whether the interview was via phone, or Zoom platform, in-person, recorded sound quality, presence of background noise, etc., played a role. On average, the accuracy was between 80 and over 90%. We added this information to the manuscript.     

**2. Results**

**2.1. How did the status of preparedness, responses, perceptions, practices, and response components vary across these countries? These should have been mentioned. **

- **Response**: The four countries were similar in terms of economic development level or health infrastructure state. As relevant to the startup, most differences were observed in the levels of availability or the shortage of local health care providers, particularly nurses, availability of qualified local vendors for outsourcing operational and logistical tasks, as well as relatively poor technological infrastructure in facilities in terms of availability of computers, internet connectivity, even electricity in some cases. The latter was partly due to the differing severity of damages caused by hurricanes that passed through the region the prior year. Hence, priorities or promptness in responsiveness varied somewhat between countries but consistent follow-up and identifying bottlenecks in a timely manner helped the team address the challenges and needs without significant delay and allow simultaneous initiation of the technical phase of the TA.  

**2.2. Triangulation with the record review/desk review has been mentioned in methods. The observations have not been mentioned clearly in the results section.**

- **Response**: Because KI typically were few in numbers representing different stakeholders and each played a unique role in the project, no one informant could provide a comprehensive picture “from the start to the end”. Therefore, triangulation or tracing and comparing data obtained from multiple sources (preliminary discussions, in-depth interviews, project documents, etc.) was done to corroborate information, construct a cohesive and accurate picture of the startup, deduce the timeline and sequence of events, or elucidate enabling and limiting factors in order to reach evaluation objectives. 

**2.3. Some narration about the results of the start-up activities should be given - whether the countries carried on the activities as planned or not.**

- **Response**: Results of the start-up phase
  The start-up activities in all four countries were conducted simultaneously except for the country visits, which were conducted sequentially by the start-up team visiting one country after the other. All four countries committed to taking on the work and to allocating the human resources needed for it. In the start-up discussions, differences between the resource availability in different countries became clear. For example, some countries had shortages in staff required to act as coaches, while others did not. These were factored into the workplan through arrangements with the Caribbean Regional Nurses Association (CRNA). By the end of the start-up phase all four countries embarked on implementing the workplan.
  The above paragraph was added to the article to address this point

**2.4. The conclusion mentions the TA as a resource-efficient model. Some information about the resource-efficiency should be mentioned.**

- **Response**: No specific cost analysis was done as part of this evaluation. The conclusion was based on the previous experience of ASSIST program leaders and TA implementers, other stakeholders, who had decades of experience in implementing similar health systems strengthening TA in multiple countries in different regions. Similar TA under normal circumstances typically involved setting up country offices, hiring local staff, and external and internal consultants, with startup activities taking much longer, often over a year, incurring significantly higher costs before TA is ready to launch.  

# Peer review: Reviewer response for version 1

**DOI:** 10.5256/f1000research.25189.r62795

**Published:** 2020-06-22

**Authors:** Lisa Hirschhorn

**Affiliations:** 1 Feinberg School of Medicine, Northwestern University, Chicago, IL, USA

The authors report on a rapid approach to provide the start-up across 4 countries in the Eastern and Southern Caribbean to begin work to improve detection of and care for children affected by Zika virus. The work is embedded in the ASSIST project, which brings deep experience in establishing quality improvement work across a range of settings, expertise which is clearly reflected in the development of this rapid approach (RMPP-MAPS) to provide short term TA reflective of the timeline of overall funding.

 The report is important in providing some important strategies for how to rapidly implement the key steps and operations needed to move rapidly, although this is relative to usual times for non-emergency interventions which would require even more rapid work (about 4 months). The strengths of the work include using an external evaluator, and the range of KIIs used. However the report could be significantly strengthened in a number of areas which need clarification.

 Methods: What, if any, frameworks were used for the qualitative coding (and KII guide development)? How were contextual factors captured and compared across the countries for similarities and differences which may have influenced implementation success of the start-up? This is described in a few areas (nurses coming from the Grenadines, coaches from existing resources versus retired nurses for example), but would be very helpful for others to be described and any association with variability of RMPP-MAPS implementation. In addition, how did ASSIST decide who to suggest for KIIs? Was there any chance of bias (should include in your limitations-which also typically go into discussion sections).

 Results and Discussion

 The scope of the evaluation is a bit confusing if this is just about start-up (as described in the results and abstract) or overall program implementation, which is described in the discussion. The authors should include information on how this approach was successful in rapid start and the outcomes of the STTA, or modify the language about the definitions of success.

 For example, in the conclusions, I do not see the relevance of the decades from research to implementation, as this was more about implementing and strengthening established EBIs? Also some data on if the EBIs were indeed implemented would strengthen the paper. Particularly given the sentence in the preceding paragraph that the broad engagement of stakeholders resulted in “promoting early institutionalization and long-term sustainability”.

 For the knowledge to be more transferable, more details of how in addition to what was done would be helpful particularly in getting leadership ownership, developing a culture for change and other areas to which ASSIST brought expertise. How did this expertise influence the success and what would another organization need to have (or not need to have) to be able to adopt the RMPP-MAPS. For example, highlighting that this was done without country offices is important for replication. What else? What partners should they choose?

 The focus on engaging a broad range of stakeholders is admirable and important. A bit more detail in how the stakeholders were identified and if the calls involving stakeholders were with all of them or more specific groups (page 11).

 The approach is described as "resource-efficient”, but no data on cost is included. That would be important as this is indeed an important factors on which TA is not always evaluated.

 Finally, the report meets many of the Consolidated criteria for reporting qualitative research (COREQ) and with a small effort would help strengthen the paper.

 Minor:

 Page 8; The authors write the approach was based on the ASSIST “model for improvement” and “adaptive management”. This needs a reference.

 Typo: Experience top of page 10 should be experienced.

Is the work clearly and accurately presented and does it cite the current literature?

Partly

If applicable, is the statistical analysis and its interpretation appropriate?

Yes

Are all the source data underlying the results available to ensure full reproducibility?

Partly

Is the study design appropriate and is the work technically sound?

Partly

Are the conclusions drawn adequately supported by the results?

Partly

Are sufficient details of methods and analysis provided to allow replication by others?

Partly

Reviewer Expertise:

Implementation science, QI, MNCH.

I confirm that I have read this submission and believe that I have an appropriate level of expertise to confirm that it is of an acceptable scientific standard, however I have significant reservations, as outlined above.

# Response

**Published:** 2022-01-04

**Authors:** M. Rashad Massoud

**Affiliations:** Department of Public Health, URC, USA

The authors report on a rapid approach to provide the start-up across 4 countries in the Eastern and Southern Caribbean to begin work to improve detection of and care for children affected by Zika virus. The work is embedded in the ASSIST project, which brings deep experience in establishing quality improvement work across a range of settings, expertise which is clearly reflected in the development of this rapid approach (RMPP-MAPS) to provide short term TA reflective of the timeline of overall funding.

 The report is important in providing some important strategies for how to rapidly implement the key steps and operations needed to move rapidly, although this is relative to usual times for non-emergency interventions which would require even more rapid work (about 4 months). The strengths of the work include using an external evaluator, and the range of KIIs used. However the report could be significantly strengthened in a number of areas which need clarification.

 Methods: What, if any, frameworks were used for the qualitative coding (and KII guide development)?

- **Response**: The case study approach and associated evaluation or learning framework guided the development of KII tools. In particular, based on initial discussions with ASSIST program officers, the evaluation focus, objectives, main topics of interest were preliminarily defined followed by identifying all stakeholders and key informants involved at each step of the startup. The interview guide was then structured to have sections around identified topics with questions and probes to facilitate detailed responses as necessary to answer evaluation questions of interest. Thematic or pattern-based coding was applied to the data manually.

How were contextual factors captured and compared across the countries for similarities and differences which may have influenced implementation success of the start-up? This is described in a few areas (nurses coming from the Grenadines, coaches from existing resources versus retired nurses for example), but would be very helpful for others to be described and any association with variability of RMPP-MAPS implementation.

- **Response**: Contextual similarities and differences were systematically identified and documented via scoping survey by the TA team during initial visits to countries. Specifically, similarities across countries in terms of socio-economic or health infrastructure development levels allowed the simultaneous and universal application of the approach. As relevant to the startup, the differences observed were more pronounced in the levels of availability of local human resources (e.g., shortage of nurses) or qualified vendors on the ground for outsourcing operational and logistical tasks. In addition, some differences were observed in the IT infrastructure readiness level, such as availability of computers, internet connectivity, or even availability of electricity in certain facilities due to differences in the destruction level caused by hurricanes that passed through the region the year before (e.g., Dominica’s health infrastructure was severely damaged compared to other countries). Therefore, appropriate specific adjustments were made to the startup planning and implementation to address any unique to specific country challenges. As indicated in the paper, to deal with these contextual differences, ASSIST integrated into its RMPP-MAPS real-time feedback mechanisms, joint brainstorming, and adaptive implementation to address challenges promptly and initiate facility-level QI activities in all four countries at the same time. 

In addition, how did ASSIST decide who to suggest for KIIs? Was there any chance of bias (should include in your limitations-which also typically go into discussion sections).

- **Response**: All individuals who were involved at any stage in the start-up were identified as KI, contacted and invited to participate in the study. The response rate was over 80%. Therefore, we believe the risk for respondent selection bias is minimal. Thank you and we included this information to the limitations which were also moved to the discussion section as suggested.

Results and Discussion

 The scope of the evaluation is a bit confusing if this is just about start-up (as described in the results and abstract) or overall program implementation, which is described in the discussion. The authors should include information on how this approach was successful in rapid start and the outcomes of the STTA, or modify the language about the definitions of success.

 For example, in the conclusions, I do not see the relevance of the decades from research to implementation, as this was more about implementing and strengthening established EBIs? Also, some data on if the EBIs were indeed implemented would strengthen the paper. Particularly given the sentence in the preceding paragraph that the broad engagement of stakeholders resulted in “promoting early institutionalization and long-term sustainability”.

- **Response**: The scope of the evaluation was limited to startup only. However, since the success of the startup phase is inevitably linked to the success of the implementation phase, we tried to mention in some form the effect on the implementation phase or the STTA itself. Regarding EBI, we removed the corresponding part on “decades of research to implementation” to avoid any confusion. We modified the language in the manuscript as shown below:

“The RMPP-MAPS model for startup facilitated a rapid launch of the implementation phase of STTA. The STTA was implemented in full scale in all four countries and engaged health workers, educators, and caregivers at all levels of the newborn and well-baby service delivery system promoting early institutionalization and long-term sustainability of the evidence-based interventions”.

 For the knowledge to be more transferable, more details of how in addition to what was done would be helpful particularly in getting leadership ownership, developing a culture for change and other areas to which ASSIST brought expertise. How did this expertise influence the success and what would another organization need to have (or not need to have) to be able to adopt the RMPP-MAPS? For example, highlighting that this was done without country offices is important for replication. What else? What partners should they choose?

- **Response**: Operating under the USAID “Journey to Self-Reliance” agenda, the ASSIST Project worked closely with government leaders to achieve locally sustained results and strengthen local capacities, with the end goal being that the work would fully transition to the government by the end of the project. This was reiterated in conversations over the life of the activity, and plans for sustainability and transition were discussed regularly. The combination of improvement expertise and upfront clarity on roles, responsibilities, and transition expectations facilitated teaming up with host country nationals and transferring competencies to them. 

The focus on engaging a broad range of stakeholders is admirable and important. A bit more detail in how the stakeholders were identified and if the calls involving stakeholders were with all of them or more specific groups (page 11).

- **Response**: Key contacts and stakeholders in the country and the region were identified in conjunction with USAID and then in conjunction with the key contacts in the country for other partners within the country.

The approach is described as "resource-efficient”, but no data on cost is included. That would be important as this is indeed an important factors on which TA is not always evaluated.

- **Response**: No specific cost analysis was done as part of this evaluation. The conclusion was based on the previous experience of ASSIST program leaders and TA implementers, other stakeholders, who had decades of experience in implementing similar health systems strengthening TA in multiple countries in different regions. Similar TA under normal circumstances typically involved setting up country offices, hiring local staff, external and internal consultants with startup activities taking much longer, often over a year incurring significantly higher costs before TA is ready to launch.  

Finally, the report meets many of the Consolidated criteria for reporting qualitative research (COREQ) and with a small effort would help strengthen the paper.

 Minor:

 Page 8; The authors write the approach was based on the ASSIST “model for improvement” and “adaptive management”. This needs a reference.

- **Response**: We added references.

Typo: Experience top of page 10 should be experienced.

- **Response**: Corrected.
